# Psychological Experiences of Patients with Coronavirus Disease 2019 (COVID-19) during and after Hospitalization: A Descriptive Phenomenological Study

**DOI:** 10.3390/ijerph19148742

**Published:** 2022-07-18

**Authors:** Yuk-Chiu Yip, Ka-Huen Yip, Wai-King Tsui

**Affiliations:** Caritas Institute of Higher Education, School of Health Sciences, 2 Chui Ling Lane, Tseung Kwan O, New Territories, Hong Kong, China; khyip@cihe.edu.hk (K.-H.Y.); ztsui@cihe.edu.hk (W.-K.T.)

**Keywords:** psychological impact, coronavirus disease 2019, descriptive phenomenological design, pulmonary rehabilitation

## Abstract

During an infectious disease pandemic, patients may experience various psychological issues. Few studies have focused on survivors’ experiences in Hong Kong. This study aimed to assess the psychological impacts of coronavirus disease 2019 (COVID-19) on survivors during admission to and discharge from COVID-19 wards using a descriptive phenomenological design. Purposive sampling was used to recruit 20 participants aged 30–77 years recently discharged from an isolation ward at an acute care facility and transferred to a community center specializing in pulmonary rehabilitation. Sampling was performed from 1 March 2022 to 3 April 2022. Semi-structured in-person interviews were conducted and transcribed verbatim; data analysis was performed using Colaizzi’s approach. The patients experienced two exclusive psychological phases during and after admission. The analysis of the patients’ experiences revealed three themes: (i) navigating uncertainties with mixed feelings and emotions during admission, (ii) adjusting to normal daily life after discharge, and (iii) self-growth after discharge. Our findings may provide empirical evidence for formulating pre-emptive strategies to mitigate the long-term psychological impacts of COVID-19. This investigation is timely and internationally relevant, and policymakers can use these findings to make informed decisions when developing guidelines for structuring the care of patients with COVID-19 during and after hospitalization. Based on our findings, we recommended that psychological support, particularly the provision of time to address patients’ concerns, may be integrated into the care of patients with COVID-19. Additionally, the structure of care may extend beyond the biomedical aspects of the illness to encompass the emotional and social dimension of the patients. To reduce stigmatization, we advise that public health authorities release clear information timely to clarify the misconceptions of the local community.

## 1. Introduction

In December 2019, the first case of pneumonia caused by severe acute respiratory syndrome coronavirus 2 (SARS-CoV-2) was reported in Wuhan, China [1,2]. The World Health Organization (WHO) officially recognized and named the disease coronavirus disease 2019 (COVID-19) [3]. The main mode of transmission of SARS-CoV-2 is through respiratory droplets when in close contact with an infected individual [4]. As COVID-19 spread rapidly throughout the world, the WHO officially declared COVID-19 a pandemic on 11 March 2020 [5].

Historically, patients have experienced adverse mental health outcomes owing to outbreaks of respiratory infectious diseases [6]. For example, patients experienced fear, anxiety, and various psychological conditions (including post-traumatic stress disorder and depression) as a result of pandemics such as SARS, Ebola, and influenza A (H1N1) [6,7,8]. COVID-19 is comparable to these pandemics in that patients experience physical, as well as psychological, issues [9]. Any such symptoms exhibited by a patient may serve as an obstacle to recovery from the disease [10]. Therefore, focus on mental health is of paramount importance during a pandemic.

Studies on the clinical and epidemiological aspects as well as the transmission method of viral diseases are continuously being conducted [11,12]. Research also includes frontline healthcare workers’ experiences whilst providing medical care to individuals diagnosed with COVID-19 [13,14]. However, there has been little research on the experiences of individuals who recovered from COVID-19 in Hong Kong [15,16,17].

To fill this knowledge gap, we aimed to investigate COVID-19 survivors’ psychological experiences during admission and discharge from isolation wards within acute care hospitals in Hong Kong, China. A qualitative approach is suitable for the nature of this study, whereby each survivor’s experience is exclusive, complex, and multidimensional, as well as personal. Such a method paves the way for detailed exploration when gathering data on the patients’ psychological experiences [18].

This study is timely and relevant to clinical practice because improved understanding of patients who have been diagnosed and subsequently recovered from COVID-19 may improve service provisions for patients and their families during and after the pandemic at both local and national levels. Healthcare workers may also gain insight into patients’ perspectives while providing care to improve strategies for sudden increases in the number of COVID-19 infections and possible future pandemics [19,20].

## 2. Materials and Methods

### 2.1. Design

This study utilized descriptive phenomenology to explore the psychological experiences of patients with COVID-19 that were admitted to and subsequently discharged from the hospital with a focus on patients’ perspectives. Husserl’s philosophy is the foundation of phenomenological research [21] and has been adopted in COVID-19-related research [13,22]. Husserl’s philosophy emphasizes the importance of understanding human consciousness by highlighting the general aspects experienced by all individuals [23]. Research thus far has provided limited information regarding the details and convolutions of the emotions and feelings arising from hospitalized patients with COVID-19. In addition, returning to normal life following discharge is regarded as a new phenomenon. Thus, descriptive phenomenology is an appropriate approach for this study in terms of fulfilling our aim to explore the general features of a phenomenon that has not yet been conceptualized in previous research [24].

Descriptive phenomenology intrinsically consists of the bracketing principle where a state of suspension exists in the defined opinions and beliefs pertaining to the studied phenomenon [21]. Bracketing reveals the underlining of the phenomenon from the perspective of the individuals that have undergone the experience. This study utilizes bracketing by adopting reflexive journaling.

Reflexive notes were kept for all stages of this study, including any preconceptions and presuppositions, researchers’ (Y.C., K.H. and W.K.) experiences in providing nursing care for patients with COVID-19 in the hospital, and the first author’s (Y.C.) thoughts immediately after the interviews were conducted. By adopting descriptive phenomenology, our research team avoided any judgmental remarks or personal interpretation and instead focused solely on elucidating the experiences from the patient’s perspective. Additionally, reflexive notes were reviewed before each interview was conducted and during data analysis in order to avoid the interviewer’s thoughts overlapping the participant’s description of their experiences.

### 2.2. Setting and Participant Recruitment

This study partnered with a community center that delivers rehabilitation services for patients with COVID-19 in Hong Kong, China, who have been recently discharged from an acute care isolation treatment facility. Purposive sampling was conducted to recruit participants within the community center with assistance from the nursing administration department from 1 March 2022 to 3 April 2022 [25]. Participants were included if they were over the age of 18 and had been admitted to an isolation ward to receive treatment related to antiviral therapy, oxygen therapy, illness monitoring, and other forms of therapy in the past two months resulting from a COVID-19 diagnosis. The participants must have been willing to discuss their experiences without language as a barrier, which means that they must have been capable of communicating in English, Cantonese, or Chinese Mandarin.

Patients were excluded from the study if they were too distressed to convey their experiences or had any form of communication barriers. The size of the sample was dependent on the principle of data saturation, where a recurrence existed in participants’ data with the absence of any new themes during the analysis [25].

### 2.3. Ethics

Participants were briefed that their participation in the study was purely voluntary. They were also informed that they reserved the right to withdraw from the study at any time, and this decision had no impact on the current care or pulmonary rehabilitation services that they were receiving from the community center at that time. Due to the highly sensitive topic, participants had access to a therapist if they showed any signs of strong emotional responses during the interview. Such access was arranged with the participant’s consent.

All participants gave written informed consent prior to the interview. Confidentiality was upheld by assigning a unique identifier for each participant instead of using their names directly when transcribing the voice data. All data in electronic form were password-protected and stored in a computer secured in a location accessible to only the researchers (Y.C., K.H. and W.K.). The raw data, which consist of audio recordings and reflexive notes, were stored at another secured location.

### 2.4. Data Collection

From 1 March 2022 to 3 April 2022, detailed face-to-face interviews were conducted using a semi-structured approach. The location of the interviews was a quiet conference room within the community center. This ensured privacy and encouraged the participants to share their experiences comfortably and openly. The interviews were conducted in person by the first (Y.C.) and second (K.H.) authors. Both the interviewers and participants wore face masks and adhered to social distancing guidelines. The duration of each interview was 55–65 min and audio recordings of the interviews were obtained. At the beginning of the interview, each participant was asked about their demographic information.

The first question was open-ended: “What was the COVID-19 admission experience like?”. The interviewer employed several prompts to encourage the participants to provide detailed responses, as shown in Table 1. The interview guide was developed through literature review [16,19,20,26,27] to ensure appropriateness considering the research aim. The interview guide was piloted by the research team (Y.C., K.H. and W.K.) with three participants, and all researchers agreed that the interview guide was appropriate, clear, comprehensive, and focused (and thus no modification was needed). Thus, the qualitative data from these three participants were also included in the final analysis. Prior to the interviews, the authors (Y.C. and K.H.) had built rapport with the participants while at the community center. Therefore, the authors (Y.C. and K.H.) were able to obtain comprehensive responses from the participants.

The first and second authors (Y.C. and K.H.) had prior experience with conducting interviews with qualitative researchers at a local university. The first author (Y.C.) has over 8 years of experience in qualitative research and has a master’s degree in nursing. The second author (K.H.) is an established qualitative researcher with a doctoral degree in health science. Therefore, the first and second authors of this study (Y.C. and K.H.) were equipped with suitable interviewing skills and were capable of employing bracketing for the entire duration of the investigation. Once the interview was completed, the authors (Y.C. and K.H.) transcribed the interviews verbatim within a day, and discussions were held with a research professor who acted as an external advisor to the research team. Where the interviews were conducted in Chinese, the transcripts were initially recorded in Chinese and then translated into English by the first author (Y.C.) and back-translated into Chinese by the third author (W.K.) to assess semantic equivalence between the original and back-translated versions of the transcripts [28]. Data collection continued until saturation, where redundant information had emerged consistently [28], which had occurred during the seventeenth interview.

### 2.5. Data Analysis

The entirety of the written and audio data was imported into the NVivo V.12 (QRS International, Burlington, MA, USA) software for data management and categorization. Colaizzi’s method of analysis was adopted, which incorporated the following seven steps. (1) Reading the transcripts three to five times in order to have a clear understanding of the conveyed message. (2) Reviewing each description and highlighting any statements that are deemed important. (3) Formulating the meaning of the important statements. (4) Categorizing the formulated meaning into thematic clusters. (5) Including the findings in an exhaustive description of the phenomenon. (6) Cross-checking the exhaustive description with the participants for validation of their emotions and/or feelings. (7) Adopting new data relevant to the final description of the phenomenon [29].

The authors (Y.C. and W.K.) coded the initial data with ongoing discussion with the previously mentioned qualitative professorial researcher for data analysis. The advisor and all the authors (Y.C., K.H. and W.K.) had mutual agreements on the final findings of the study. The authors (Y.C., K.H. and W.K.) adhered to the Consolidated Criteria for Reporting Qualitative Research guidelines for the entire duration of this study [30].

### 2.6. Rigor

The study’s trustworthiness was elevated by adopting Lincoln and Guba’s evaluative criteria for qualitative research [31]. The credibility of this study was supported via the implementation of bracketing for the entire duration of the study while excluding any personal knowledge and experience of the phenomenon. Furthermore, close rapport was built with each participant, and the participants were subsequently intensely engaged to obtain the data. The transferability was maintained throughout by the provision of comprehensive descriptions of participants’ experiences with relevant citations of their verbatim statements.

Dependability was ascertained through clear audit trails of the method by which analytic decisions were made. Confirmability was ensured through the sharing of essential themes and sub-themes with the participants. The first author (Y.C.) also established member-checking by providing the participants with exhaustive descriptions of the phenomenon. All participants agreed that the exhaustive descriptions reflected the true value of their experiences as a COVID-19 patient admitted and subsequently discharged from the hospital.

## 3. Results

Twenty-one individuals were approached by the research team. Only one of them (male) declined the invitation to participate in this study because he perceived the experiences as “quite upset to share with others”. The sample ultimately consisted of 20 individuals aged 30 to 77 years (11 women and 9 men). With the adoption of purposive sampling, a heterogeneous sample consisting of individuals that were previously diagnosed with COVID-19 as well as experienced medical treatment while admitted to an acute care facility was selected. Eight participants were considered index cases; that is, they were the first individuals within their family to test positive for COVID-19 using nucleic acid testing or a self-administered antigen test. Twelve participants were married. These individuals had diverse background characteristics including age, sex, occupation, and marital status. Table 2 shows the background characteristics of the participants.

The patients who were diagnosed with COVID-19 went through two distinct phases of psychological experiences during admission and after discharge from the hospital. The analysis showed three themes: (1) experiencing uncertainties with mixed feelings and emotions while being admitted, (2) attempting to return to normal life after being discharged, and (3) self-growth after being discharged.

### 3.1. Theme 1: Navigating Uncertainties with Mixed Feelings and Emotions during Hospitalization

#### 3.1.1. Searching for an Explanation for the Infection

Participants reported that when they were admitted, they frequently thought about the possible route of infection. This may be unrelated to their scientific understanding of COVID-19 transmission. As such, participants often made their own conclusions regarding how they became infected and attempted to reflect on the preventative measures that they could have taken to avoid becoming infected:

“I was very conscious of my hand hygiene, I wear KF94 [KF denotes Korean Filter and 94 denotes filtration efficacy] masks whenever I went outside. I still got infected with this disease and can’t help but ask myself, ‘what did I miss?’”.

(P6, male)

“I understand that this disease is airborne, but I practiced social distancing and wore a mask at all times. I frequently ask myself how I became infected and what I missed [infection control measure]”.

(P9, female)

During the first few days of admission, participants attempted to figure out how they became infected. When they found out how they possibly became infected, they felt some relief, which was a psychological anchor that they discovered. Such an anchor alleviated their negative experiences:

“By thinking back and understanding it, at least I have some explanation as to how I became infected… (sighs). Maybe I just needed some form of explanation to make sense of all this [infection]. I was totally shocked when I tested positive in the nucleic acid test”.

(P1, male)

#### 3.1.2. Experiencing Psychological Distress as an In-Patient

Participants who had been admitted to the isolation wards reported feeling confused for the duration of the medical treatment. The majority of them said that the confusion stemmed from a spike in admissions as a result of the Omicron variant, which resulted in enormous pressure upon the public health service. The realization of the actual situation made the patients feel powerless, in addition to the high-pressure nature of the ward environment where healthcare professionals consisting of both nurses and physicians had to go above and beyond to provide medical care according to the patients’ needs.

“I felt powerless when I was lying on the bed while being treated with intravenous infusion as well as wearing an oxygen mask. I could see that the nurses, physicians, and other healthcare professionals were rushing about [the isolation ward]. Medical equipment alarms were blaring all over the place simultaneously with moans from other patients. It was a very stressful situation for me”.

(P8, female)

The majority of participants reported difficulty in accessing nurses and physicians with the hope of addressing their concerns. These participants felt helpless as they were unsuccessful in receiving information on their current status from the health professionals:

“I appreciate the fact that nurses were too preoccupied with their nursing tasks. It became a luxury for us as patients to be able to get their attention for just a short period of time to discuss the treatment plan…I was facing multiple uncertainties as I have little knowledge of medicine and the nature of the infection”.

(P2, female)

“…helplessness would be the right term to explain what I was feeling. The nurses and physicians have to work very hard. But we, as patients, still desperately need information related to the therapeutic effect after being administered medication….at the end of the day, any form of reassurance, regardless of how little, would have been good”.

(P4, female)

The most important information to alleviate anxiety during the hospitalization that could be obtained from healthcare professionals from the participants’ perspective was related to the impact of the disease, treatment plan, and updates on their conditions. Many participants were most concerned with the long-term implications of COVID-19 infection related to their comorbidities.

“For 17 years I have been living with diabetes and only two years ago, I had to undergo cardiac catheterization. Are there further complications from COVID-19 due to my diabetes treatment? I feel depressed due to the uncertain nature of the answers given by the nurses and the physicians”.

Participants expressed that they felt guilty as they were left wondering if their health status had a negative impact on their family members such as children that lived with them, colleagues, and frequent social contacts. They felt they had become an “agent of danger” in their community, whereby they posed a health threat to a large number of individuals. Their feeling stemmed from the possibility of them infecting others due to Omicron’s high infectivity.

“As we know from the experts [university professors and physicians] that appeared on TV, a person can infect others before he/she realizes that he/she is COVID-19-positive… I felt guilty because my 86-year-old mother lives with me and she was also diagnosed with this disease. Even though she did not blame me at all, I am certain I infected her because I was having headaches and sore throat three days before my mother also started displaying the same symptoms… I feel really bad for infecting anyone that came physically close to me”.

(P9, female)

“For the first two days, I cried many times during admission… I felt that if my 3-year-old daughter was also diagnosed with the disease, it would totally be on me”.

(P2, female)

“I would be okay if the disease only impacted me. I would feel sorry if I was the individual that spread the disease to other people in the community”.

(P3, male)

Some participants had both fear and anger as they became increasingly worried about the pandemic. They were angry about the lack of healthcare resources as well as the lack of attention to patients’ concerns and needs by healthcare professionals (physicians and nurses).

“The monitors in the medical rooms were very noisy…I asked a nurse if something could be done about it because patients need to rest…The nurses ignored me and simply brushed me off by saying ‘We [nurses] have to monitor the patients. The alarms coming from these monitors are important when something is seriously wrong.’ I was angry as patients’ basic rights to rest and sleep were not protected”.

(P8, female)

The participants experienced fear on a gradual basis during the admission process after being informed of their family and friends being infected and hearing the news on TV. Most participants had psychological distress after knowing the latest information as the majority of it consisted of negative news about the pandemic.

“Some friends assured me that the symptoms of patients diagnosed with COVID-19 were mild… but… I was scared when the news reported deaths, which were increasing getting each day”.

(P1, male)

“I watched the news on the internet about new complications that were just becoming known, such as brain fog…and I was worried because I did not know that acute infection may result in long-term complications”.

(P5, male)

An overwhelming amount of information from the internet was the major cause of psychological distress, as expressed by all participants. The participants understood the power of the internet in broadcasting information, which may be accurate or false. Furthermore, such information can be transmitted to various social media apps including WeChat, WhatsApp, and Twitter. Participants were distressed psychologically when attempting to determine if particular information was accurate as they had no medical knowledge.

“My mobile phone was next to me when I was in the hospital. I was in several groups on WeChat and WhatsApp…I get my news from media outlets and online messages, all messages including those that are true, false, positive, and negative. Such messages impacted my understanding of the disease and changed my thoughts on the [medical] situation that I was facing at that time”.

(P17, female)

### 3.2. Theme 2: Adjusting Back to Normal Daily Life after Being Discharged

#### 3.2.1. Making Up for Lost Time with Family Reunification

Prior to being hospitalized, the participants stated that they took on many roles in their families, including caring for parents, children, and spouses. This situation changed once they were placed in isolation wards, and their family duties were halted. Once their hospitalization ended, the majority of participants focused on rebuilding their familial bonds, and they felt they needed to make up for the time spent apart from their families.

“I have been isolated in hospital for over 15 days because of COVID, and I hated being stuck in that environment with no opportunity to go outside. Normally, my wife and I go hiking in Sai Kung, but I missed this when I was in hospital. I plan to hike with my family in the New Territories, and I intend to maintain this a habit in the future as my familial bonds are so important to me”.

(P13, male)

#### 3.2.2. Living with Gratitude

Even though they faced the dangerous prospect of dealing with a potentially life-threatening virus, certain individuals were extremely mentally resilient towards the illness. It was even mentioned that COVID-19 encouraged them to pursue personal growth, and it motivated them to lead healthier lives in the future.

“Prior to contracting COVID, I felt my family wanted me to work harder for them, and that this would bring them happiness, so I did not give much weight to my physical health. When I was in hospital, I had time to reflect on my choices and understood that I could not overcome the difficulties of the pandemic without my family’s support. I feel blessed to have received their love throughout this period”.

(P16, male)

#### 3.2.3. Facing Social Judgment

Even after they had been discharged from hospital, the participants felt that they were discriminated against when they initially returned to their communities because of their recent infection by members of the public who had not been infected before:

“Even though I was medically fit for work, my supervisor still requested that I do not return to the office for 14 days after I left hospital. This is because he was told certain cases remain infectious after being cleared from hospital, and even though the chances of spreading the virus were low, he did not want the responsibility of any such consequences”.

(P18, male)

“My husband’s parents insisted that I do not go to their family dinner because of their age and wanting to be extra cautious. I was told not to visit for at least a month, but this felt like I was discriminated against because I had a slim possibility of being contagious. I believe my parents-in-law were not confident in my state of health at that time”.

(P7, female)

#### 3.2.4. Living with Economic Stress

The public health system in Hong Kong suffered greatly from the fifth COVID-19 wave, as infections surpassed one million cases. Participants stated that the social distancing measures were strict, including bans on evening restaurant dining and closures of bars and other leisure venues. Small and medium-sized businesses and socioeconomic structures in Hong Kong were faced with major hurdles due to the fifth wave of the pandemic, as discussed by one participant:

“Previously, I would deliver take-out food on a daily basis, as a self-employed worker. I do not have medical insurance, so when I became sick and was treated at the hospital, my income was reduced to zero. Once released from hospital, restaurants were closed due to the evening dining ban in place after 6 pm. I was unable to support my family at this time, and I felt distressed at the situation”.

(P20, male)

While the government paid for the base medical costs incurred by COVID-19, patients were still under strong financial pressure. They felt lost after their discharge from hospital, and had to manage new, difficult conditions without any extra support.

“I am the breadwinner of my family, but since I was released from hospital, I struggled to cover all our expenses as I was not earning any money when I was in hospital. My employer dismissed me from my job during my hospitalization because their business had started to fail. I did not have enough to pay for my family in my bank account and I could not pay my rent, and I felt ignored by the government at this time”.

(P10, male)

### 3.3. Theme 3: Self-Growth after Being Discharged

#### 3.3.1. Appreciation of the Received Social Support

Participants felt love and support from a number of sources, including family, friends, and healthcare professionals in charge of dealing with COVID-19 in Hong Kong. Participants were very sympathetic and appreciative towards the medical staff, who faced great risks in their efforts to control the pandemic and protect the health of the public.

“Healthcare workers offered care to their patients even though this posed a risk to their own health, and they worked hard despite difficult conditions (smile)”.

It was also stated that the Chinese government medical and nursing staff offered vital assistance to the city of Hong Kong during the fifth wave of COVID-19. Participants felt that China’s support at the macro level was of great help to the people of Hong Kong.

“I was glad to have the unconditional support of my country during the fifth wave, and I feel that without it, Hong Kong would have had a much higher death and infection rate. The frontline healthcare workers involved have my endless gratitude”.

#### 3.3.2. Becoming a Proactive Citizen to Participate in Whole-of-Society Response

Participants who were hospitalized with COVID endured substantial feeling of stress. They felt they were failing in their social and family roles, and were no longer able to look after their loved ones. Many participants attempted to convince those around them to become vaccinated, and they openly discussed their negative experiences in order to motivate their friends and family to become vaccinated.

“I feel indebted to society, as the Hong Kong medical system took such good care of me. After my recovery, I felt the urge to tell my loved ones about my recent experiences, and actively try to help the government in their efforts to control the pandemic by persuading those around me to get vaccinated, which is a key step”.

(P14, female)

Family, friends, and healthcare workers were described as offering vital support during participant hospitalization. This helped increase the awareness participants had of their social role and responsibilities as Hong Kong citizens. Moreover, these individuals felt strongly about adhering to the government guidelines regarding social distancing and COVID-19 testing, in order to reduce the pandemic’s effects.

“After I caught COVID, the doctors and nurses caring for me made a huge impression on me, and I feel very grateful for their help throughout the rehabilitative period. Once I recovered, my outlook on life changed, and I started to actively try to motivate people to follow government guidelines, and I felt obliged to pay back society”.

(P6, male)

## 4. Discussion

This study conducted a qualitative examination of the psychological experiences patients with COVID-19 had at the time of their hospitalization in an acute care facility, and once they were released from an isolation ward. The findings denoted that patients were under severe stress, stemming from the disease, treatment information they were infrequently given by healthcare professionals, and the isolation ward’s hectic environment, as well as the discrimination they felt once they rejoined society after being discharged. Although participants had to deal with social prejudice and economic difficulties, these now-healthy individuals experienced support from many sources, including their loved ones and the Chinese government. They felt very grateful for all the care they received, and in some cases, survivors became more aware of their responsibilities and became more mature through these experiences. These individuals began to actively take part in their community’s fight against COVID-19. This qualitative study offers insight into the psychological implications of the viral infection from many perspectives, which can assist in finding ways to alleviate psychological issues and stress in the future.

Patients with COVID-19 initially reported fear, anger, feelings of uncertainty, confusion, powerlessness, and guilt once they were hospitalized. The results of this paper showed that mental distress was a key issue throughout the COVID-19 pandemic, which echoed the findings of existing studies [32]. The current study also found that there are many negative experiences associated with COVID-19 for patients, who had a growing number of concerns and stresses [33,34]. Most of the emotional distress is potentially because of the contagious nature of COVID-19, the uncertainty surrounding infection, limited information about the disease, and the antiviral therapeutics countering COVID-19 [35,36]. In turn, patients felt worried about the disease and how it related to them. Individuals with comorbidities were shown to suffer higher levels of stress regarding COVID-19 compared to other patients [37]. In addition, information was spread much faster and wider during this pandemic compared to the SARS epidemic of 2003 [38], which led to a degree of misinformation, as confirmed in this paper. A quantitative study by Zhong et al. [39] showed that social media overuse was closely correlated with depression and secondary trauma. Disease statistics and prevention methods were among the topics of misinformation spread across popular social media platforms [40]. The WHO described this situation as the “coronavirus infodemic” [41], and it has induced emotional distress, worry, fear, and traumatic outbursts [42,43]. Thus, this “infodemic” must be accounted for when trying to assist patients with COVID-19 who are emotionally distressed.

Once they left their isolation wards, participants felt a different set of psychological experiences after they attempted to return to their community and their daily routines. We found that these individuals wanted to account for the time spent away from their families after discharge, and they felt pressure to meet their familial responsibilities while simultaneously dealing with social prejudice and poor economic conditions. These negative psychological experiences brought about feelings of disappointment in participants [26]. The concerns they felt regarding social discrimination are referred to as COVID-19-related stigma, and this is a vital aspect of the conceptual framework of health-related quality of life in previously hospitalized patients with COVID-19 [44]. These social prejudices were primarily caused by misinformation and a lack of education regarding the pandemic [45]. The result was significant psychosocial hurdles and anxiety being more widespread [46], which acted as a barrier for recently infected individuals to seek help and receive an official COVID-19 diagnosis and treatment [27,32]. Thus, the apparent stigma towards COVID-19 infection must be overcome in order to allow for discharged patients to fully rejoin their communities. Poor financial security was another key contributor towards the deterioration of patients’ mental health. This was also seen in research conducted during the recovery period after the SARS epidemic, where financial worries were the leading psychological distress [47]. Notably, migrant workers who caught COVID-19 in Singapore did not describe any anxiety about economic conditions [48]. This was potentially because these workers continued to be paid throughout their quarantine, which is a measure worthy of inclusion in future policy decisions.

Once released from the hospital and readjusting to their everyday lives, patients expressed gratitude at the widespread social support they received from many sources, including family members, colleagues, healthcare professionals, and the Chinese government. Research has found that social support is a critical protective element for a person’s mental health following their recovery from a stressful incident [49]. In addition, patients felt more relaxed and recovered faster if they were offered sufficient social support [50,51]. The current paper denotes that patients were more aware of their social obligations following their experiences. A possible cause for this is the Chinese culture, which is collectivism-oriented and is closely tied with the Confucian tradition of morality [52]. Under Chinese culture, altruism and looking after other members of society are values instilled in all citizens. Following their COVID-19 experiences, participants felt they needed to be more active in their efforts to persuade their social circles to become vaccinated, in order to protect those around them. They felt obliged to motivate other people to adhere to government guidelines against the fifth wave of the pandemic in Hong Kong. These results are in line with other studies on the topic [14]. Conversely, greater awareness of social obligations and religion were not shown to have a clear correlation in this study, which is different from the findings of some other studies [48,50].

### 4.1. Strengths

The current paper investigated the psychological experiences of patients with COVID-19 in Hong Kong during the fifth wave of the pandemic, and is the first of its kind. There are limited existing qualitative studies examining related topics [16,45]. This study’s findings enrich the existing understanding of the psychological experiences that patients go through during and after hospitalization due to COVID-19, particularly in the Chinese context. In turn, our data can help guide the approaches taken to deal with this type of emotional distress. Detailed descriptions of these experiences were collected using a phenomenological approach, which aimed to understand the deeper impact of the lived experience. In turn, inductive thematic analysis conducted with line-by-line open coding and allowing interviewees to speak openly is the ideal methodology for such a phenomenological approach. Furthermore, observations and field notes gathered throughout the interviews act as additional sources of information for the researcher.

### 4.2. Limitations

This study had some limitations. The inclusion criterion of no language barriers was subjectively decided by the author, since a lack of time, funding, and necessary resources did not allow the study to be more objective and in-depth in its testing. On the other hand, it is recommended that each study use sampling and data collection methods of suitable complexity for its design to maintain the feasibility of its resources [53]. Second, the study context was Hong Kong, and results from other locations may differ due to disparities in isolation facility environments, geographical locations, and cultures. The sample involved in this study was made up of patients who suffered from mild to moderate clinical symptoms, therefore reducing the generalizability of the results to wider COVID-19 patient populations. Notably, for future research, patients with varying levels of illness should be included. Another limitation is the potential validity of the results, even though the researchers made great efforts to reduce presuppositions. We observed that participants were highly grateful towards nurses, and this might be because the author is a nurse by profession. Thus, it was necessary to remind participants that their experiences were the focal point of this study, as well as the impact these experiences had on them. Lastly, our research team decided to exclude patients who presented with emotional distress (for example, those who appeared overly perturbed in recalling or sharing the illness experience and recovery process of COVID-19). The decision was based on our ethical consideration for the protection of mental integrity of these individuals. Professionally, as both nurse scientists and advanced practitioners, we recognized that some of the experiences from these individuals may have been left unrecorded. We thus recommended that clinical psychologists may be involved to be one of the interviewers as they can assist in managing unpleasant emotional responses during and/or after an interview.

### 4.3. Relevance to Clinical Practice and Implications

This paper underlined the fact that patients with COVID-19 required not only medical care for their physical illness, but also emotional support. Psychosocial stresses include comorbidities, social stigma, socioeconomic struggles, and misinformation on social media, as defined in research in other countries as well [40,54]. In order to manage the feelings of doubt, concern, and stress in patients, healthcare professionals are encouraged to educate patients on the therapy options they have against COVID-19, along with updates on their health condition, in order to clarify their illness perception and alleviate mental turmoil through patient empowerment [55,56]. In addition, psychological reviews and short support sessions are advised on a frequent basis through virtual means, and staff who come into frequent contact with patients with COVID-19 should learn the early signs and symptoms of mental health problems in order to begin early interventions. These healthcare workers can refer patients to mental health experts in more severe cases, while the patients themselves should be advised to talk with family and friends through video or text conversations and receive psychological support. It was noted that COVID-19 survivors felt rejected and stigmatized following their recovery by suspicious members of the public who believed the patients were still contagious, as described by participants of the current study. In order to bring about a smoother return to society, the public must be educated about the psychological needs of those returning from isolation, and the fact that these survivors are no longer contagious must be made clear. It is vital that accurate information is shared about the status of COVID-19 by public health authorities, while employment guarantees are made by local governments. A positive outlook is a crucial component of COVID-19 survivors’ psychological recovery [57,58]. It should be the responsibility of frontline healthcare professionals to offer appropriate psychological interventions for patients in order to inspire positive emotions and alleviate psychological distress [59].

## 5. Conclusions

There have been drastic consequences to the rapid spread of COVID-19 across the globe, and this study attempted to examine the psychological experiences of hospitalized patients with COVID-19, along with their feelings and/or emotions when returning to their local communities following discharge. In this study, the complicated psychological experiences patients had included fear, anger, powerlessness, confusion, and guilt. These emotions and feelings were stronger in the stressful ward environment, as there was a lack of information shared regarding the disease and physical status of the patients. There was not enough time given to answer the patients’ questions, and once they were released from the hospital, patients struggled to readjust to their daily lives due to social stigma. Some patients went through a period of self-growth after their illness, feeling more positively towards society and the Chinese government, as well as their social obligations. The results of this study aim to guide the development of tailored intervention programs, thereby reducing instances of long-term negative psychological consequences in patients. In addition, investigating such a recent pandemic event produces vital evidence-based data which can assist in future guideline-setting related to the provision of psychosocial care for patients with COVID-19 overseas. Further studies may focus on the exploration of suitable psychological interventions aimed at instilling positive emotions in patients, alleviating negative emotional states and feelings, and protecting their mental wellbeing.

## Figures and Tables

**Table 1:** Interview guide.

No.	Probing Questions
1.	Can you tell me about your overall experience after you were confirmed to have COVID-19?
2.	How did you feel when you were hospitalized in an isolation ward (where, due to the fifth wave of the COVID-19 pandemic, family visits were not allowed)?
3.	How did you feel when you completed the in-hospital treatment and were deemed medically fit for discharge?
4.	When you rejoined the community following hospitalization, what were the positive and negative experiences?
5.	Reflecting on your experience through the infection, treatment, and recovery in this fifth wave of the COVID-19 pandemic, how have your views on your future life and the society changed?

COVID-19, coronavirus disease 2019.

**Table 2:** General characteristics of participants (*N* = 20).

Variables	Characteristics	*N* (%)
Age, year	30–39	4 (20)
	40–49	9 (45)
	50–59	5 (25)
	≥60	2 (10)
Sex	Male	9 (45)
	Female	11 (55)
Marital status	Single	6 (30)
	Married	12 (60)
	Divorced	2 (10)
Occupation	Employed	12 (60)
	Unemployed	8 (40)
Index case ^1^	Self	8 (40)
	Partner	5 (25)
	Mother	2 (10)
	Father	3 (15)
	Mother-in-law	1 (5)
	Father-in-law	1 (5)

^1^ First patient that tested positive in a family cluster.

## Data Availability

The interview guide is provided in the manuscript using a table. The transcripts that contain private and confidential data, such as the isolation wards and hospitals in which the participants were admitted, the service provider, rehabilitation services, and duration of pulmonary rehabilitation will not be made publicly available, to protect the participants’ privacy.

## References

[1] Lu H., Stratton C.W., Tang Y.W. (2020). Outbreak of pneumonia of unknown etiology in Wuhan, China: The mystery and the miracle. J. Med. Virol..

[2] Coronaviridae Study Group of the International Committee on Taxonomy of Viruses (2020). The species severe acute respiratory syndrome-related coronavirus: Classifying 2019-nCoV and naming it SARS-CoV-2. Nat. Microbiol..

[3] WHO Director-General’s Remarks at the Media Briefing on nCoV on 11 February 2020. https://www.who.int/dg/speeches/detail/who-director-general-s-remarks-at-the-media-briefing-on-2019-ncov-on-11-february-2020.

[4] How COVID-19 Spreads. www.cdc.gov/coronavirus/2019-ncov/prevent-getting-sick/how-covid-spreads.html?CDC_AA_refVal=https%3A%2F%2Fwww.cdc.gov%2Fcoronavirus%2F2019-ncov%2Fprepare%2Ftransmission.html.

[5] WHO Director-General’s Opening Remarks at the Media Briefing on COVID-19—11 March 2020. https://www.who.int/dg/speeches/detail/who-director-general-s-opening-remarks-at-the-media-briefing-on-covid-19%2D%2D-11-march-2020.

[6] Shah K., Kamrai D., Mekala H., Mann B., Desai K., Patel R.S. (2020). Focus on mental health during the coronavirus (COVID-19) pandemic: Applying learnings from the past outbreaks. Cureus.

[7] Chew Q.H., Wei K.C., Vasoo S., Chua H.C., Sim K. (2020). Narrative synthesis of psychological and coping responses towards emerging infectious disease outbreaks in the general population: Practical considerations for the COVID-19 pandemic. Singapore Med. J..

[8] Bo H.X., Li W., Yang Y., Wang Y., Zhang Q., Cheung T., Wu X., Xiang Y.T. (2021). Posttraumaticstress symptoms and attitude toward crisis mental health services among clinically stable patients with COVID-19 in China. Psychol. Med..

[9] Shanbehzadeh S., Tavahomi M., Zanjari N., Ebrahimi-Takamjani I., Amiri-Arimi S. (2021). Physical and mental health complications post-COVID-19: Scopingreview. J. Psychosom. Res..

[10] Heymann D.L., Shindo N., WHO Scientific and Technical Advisory Group for Infectious Hazards (2020). COVID-19: What is next for public health?. Lancet.

[11] Chan J.F.-W., Yuan S., Kok K.H., To K.K., Chu H., Yang J., Xing F., Liu J., Yip C.C., Poon R.W. (2020). A familial cluster of pneumonia associated with the 2019 novel coronavirus indicating person-to-person transmission: A study of a family cluster. Lancet.

[12] Song R., Han B., Song M., Wang L., Conlon C.P., Dong T., Tian D., Zhang W., Chen Z., Zhang F. (2020). Clinical and Epidemiological Features of COVID-19 Family Clusters in Beijing, China. J. Infect..

[13] Sadang J.M. (2021). The lived experience of Filipino nurses’ work in COVID-19 quarantine facilities: A descriptive phenomenological study. Pac. Rim Int. J. Nurs. Res..

[14] Liu Q., Luo D., Haase J.E., Guo Q., Wang X.Q., Liu S., Xia L., Liu Z., Yang J., Yang B.X. (2020). The experiences of health-care providers during the COVID-19 crisis in China: A qualitative study. Lancet Glob. Health.

[15] Tsamakis K., Tsiptsios D., Ouranidis A., Mueller C., Schizas D., Terniotis C., Nikolakakis N., Tyros G., Kympouropoulos S., Lazaris A. (2021). COVID-19 and Its Consequences on Mental Health [Review]. Exp. Ther. Med..

[16] Moradi Y., Mollazadeh F., Karimi P., Hosseingholipour K., Baghaei R. (2020). Psychological disturbances of survivors throughout COVID-19 crisis: A qualitative study. BMC Psychiatry.

[17] Ladds E., Rushforth A., Wieringa S., Taylor S., Rayner C., Husain L., Greenhalgh T. (2020). Persistent symptoms after COVID-19: Qualitative study of 114 ″long COVID” patients and draft quality principles for services. BMC Health Serv. Res..

[18] Teti M., Schatz E., Liebenberg L. (2020). Methods in the time of COVID-19: The vital role of qualitative inquiries. Int. J. Qual. Methods.

[19] Schwerdtle P.M., De Clerck V., Plummer V. (2017). Experiences of Ebola survivors: Causes of distress and sources of resilience. Prehosp. Disaster Med..

[20] Shaban R.Z., Nahidi S., Sotomayor-Castillo C., Li C., Gilroy N., O’Sullivan M.V.N., Sorrell T.C., White E., Hackett K., Bag S. (2020). SARS-CoV-2 infection and COVID-19: The lived experience and perceptions of patients in isolation and care in an Australian healthcare setting. Am. J. Infect. Control.

[21] Dowling M. (2007). From Husserl to Van Manen. A review of different phenomenological approaches. Int. J. Nurs. Stud..

[22] Karimi Z., Fereidouni Z., Behnammoghadam M., Alimohammadi N., Mousavizadeh A., Salehi T., Mirzaee M.S., Mirzaee S. (2020). The lived experience of nurses caring for patients with COVID-19 in Iran: A phenomenological study. Risk Manag. Healthc. Policy.

[23] Lopez K.A., Willis D.G. (2004). Descriptive versus interpretive phenomenology: Their contributions to nursing knowledge. Qual. Health Res..

[24] Wojnar D.M., Swanson K.M. (2007). Phenomenology: An exploration. J. Holist. Nurs..

[25] Moser A., Korstjens I. (2018). Series: Practical guidance to qualitative research. Part 3: Sampling, data collection and analysis. Eur. J. Gen. Pract..

[26] Hsiao C.T., Sun J.J., Chiang Y.H., Chen H.L., Liu T.Y. (2021). Experience of patients with COVID-19 in hospital isolation in Taiwan. Nurs. Health Sci..

[27] Rahmatinejad P., Yazdi M., Khosravi Z., Shahisadrabadi F. (2020). Lived experience of patients with coronavirus (COVID-19): A phenomenological study. J. Res. Psychol. Health.

[28] Polit D.F., Beck C.T. (2017). Nursing Research: Generating and Assessing Evidence for Nursing Practice.

[29] Colaizzi P.F., Valle R.S., King M. (1978). Psychological Research as the Phenomenologist Views It. Existential-Phenomenological Alternatives for Psychology.

[30] Tong A., Sainsbury P., Craig J. (2007). Consolidated Criteria for Reporting Qualitative Research (COREQ): A 32-item checklist for interviews and focus groups. Int. J. Qual. Health Care.

[31] Lincoln Y.S., Guba E.G., Pilotta J.J. (1985). Naturalistic nquiry. Int. J. Intercult. Relat..

[32] Alipour F., Arshi M., Ahmadi S., LeBeau R., Shaabani A., Ostadhashemi L. (2020). Psychosocial challenges and concerns of COVID-19: A qualitative study in Iran. Health.

[33] Foli K.J., Forster A., Cheng C., Zhang L., Chiu Y.C. (2021). Voices from the COVID-19 frontline: Nurses’ trauma and coping. J. Adv. Nurs..

[34] Akkuş Y., Karacan Y., Güney R., Kurt B. (2022). Experiences of nurses working with COVID-19 patients: A qualitative study. J. Clin. Nurs..

[35] Lima C.K.T., Carvalho P.M.M., Lima I.A.A.S., Nunes J.V.A.O., Saraiva J.S., de Souza R.I., da Silva C.G.L., Neto M.L.R. (2020). The emotional impact of coronavirus 2019-nCoV (new coronavirus disease). Psychiatry Res..

[36] Xiang Y.T., Yang Y., Li W., Zhang L., Zhang Q., Cheung T., Ng C.H. (2020). Timely mental health care for the 2019 novel coronavirus outbreak is urgently Needed. Lancet Psychiatry.

[37] Wang C., Pan R., Wan X., Tan Y., Xu L., Ho C.S., Ho R.C. (2020). Immediate psychological responses and associated factors during the initial stage of the 2019 coronavirus disease (COVID-19) epidemic among the general population in China. Int. J. Environ. Res. Public Health.

[38] Wong J.E.L., Leo Y.S., Tan C.C. (2020). COVID-19 in Singapore-current experience: Critical global issues that require attention and action. JAMA.

[39] Zhong B., Huang Y., Liu Q. (2021). Mental health toll from the coronavirus: Social media usage reveals Wuhan residents’ depression and secondary trauma in the COVID-19 outbreak. Comput. Hum. Behav..

[40] Bastani P., Bahrami M.A. (2020). COVID-19 related misinformation on social media: A qualitative study from Iran. J. Med. Internet Res..

[41] Zarocostas J. (2020). How to fight an infodemic. Lancet.

[42] Allahverdipour H. (2020). Global challenge of health communication: Infodemia in the coronavirus disease (COVID-19) pandemic. J. Educ. Commun. Health.

[43] Hua J., Shaw R. (2020). Corona virus (COVID-19) “infodemic” and emerging issues through a data lens: The case of China. Int. J. Environ. Res. Public Health.

[44] Wu C., Cheng J., Zou J., Duan L., Campbell J.E. (2021). Health-related quality of life of hospitalized COVID-19 survivors: An initial exploration in Nanning City, China. Soc. Sci. Med..

[45] Liu W., Liu J. (2021). Living with COVID-19: A phenomenological study of hospitalised patients involved in family cluster transmission. BMJ Open.

[46] Mak W.W., Mo P.K., Cheung R.Y., Woo J., Cheung F.M., Lee D. (2006). Comparative stigma of HIV/AIDS, SARS, and tuberculosis in Hong Kong. Soc. Sci. Med..

[47] Mihashi M., Otsubo Y., Yinjuan X., Nagatomi K., Hoshiko M., Ishitake T. (2009). Predictive factors of psychological disorder development during recovery following SARS outbreak. Health Psychol..

[48] Yee K., Peh H.P., Tan Y.P., Teo I., Tan E.U.T., Paul J., Rangabashyam M., Ramalingam M.B., Chow W., Tan H.K. (2021). Stressors and coping strategies of migrant workers diagnosed with COVID-19 in Singapore: A qualitative study. BMJ Open.

[49] Postigo-Zegarra S., Julián M., Schoeps K., Montoya-Castilla I. (2021). psychological adjustment of Spanish adolescents and their parents during COVID-19 lockdown: A mixed method approach. PLoS ONE.

[50] Jamili S., Ebrahimipour H., Adel A., Badiee Aval S., Hoseini S.J., Vejdani M., Ebnehoseini Z. (2022). Experience of patients hospitalized with COVID-19: A qualitative study of a pandemic disease in Iran. Health Expect..

[51] Karimi L., Makvandi S., Rahimi-Bashar F., Vahedian-Azimi A., Sahebkar A. (2021). The experiences of recovered COVID-19 patients in Baqiyatallah hospital: A qualitative study. Adv. Exp. Med. Biol..

[52] Cheng S.K. (1990). Understanding the culture and behaviour of East Asians—A Confucian perspective. Aust. N. Z. J. Psychiatry.

[53] Ayres L. (2007). Qualitative research proposals--part iii: Sampling and data collection. J. Wound Ostomy Cont. Nurs..

[54] Akbarbegloo M., Sanaeefar M., Majid P., Mohammadzadeh M. (2022). Psychosocial care experiences of patients with COVID-19 at home in Iran: A qualitative study. Health Soc. Care Community.

[55] Chevance A., Gourion D., Hoertel N., Llorca P.-M., Thomas P., Bocher R., Moro M.-R., Laprévote V., Benyamina A., Fossati P. (2020). Ensuring mental health care during the SARS-CoV-2 epidemic in France: A narrative review. L’Encéphale.

[56] Halvorsen K., Dihle A., Hansen C., Nordhaug M., Jerpseth H., Tveiten S., Joranger P., Knutsen I.R. (2020). Empowerment in healthcare: A thematic synthesis and critical discussion of concept analyses of empowerment. Patient Educ. Couns..

[57] Waugh C.E. (2020). The roles of positive emotion in the regulation of emotional responses to negative events. Emotion.

[58] Carbone E.G., Echols E.T. (2017). Effects of optimism on recovery and mental health after a tornado outbreak. Psychol. Health.

[59] Duan L., Zhu G. (2020). Psychological interventions for people affected by the COVID-19 epidemic. Lancet Psychiatry.

